# Expression profiling of laser-microdissected intrapulmonary arteries in hypoxia-induced pulmonary hypertension

**DOI:** 10.1186/1465-9921-6-109

**Published:** 2005-09-19

**Authors:** Grazyna Kwapiszewska, Jochen Wilhelm, Stephanie Wolff, Isabel Laumanns, Inke R Koenig, Andreas Ziegler, Werner Seeger, Rainer M Bohle, Norbert Weissmann, Ludger Fink

**Affiliations:** 1 Department of Pathology, Justus-Liebig-University Giessen, Germany; 2 Department of Medical Biometry and Statistics, University at Luebeck, Germany; 3 Department of Internal Medicine, Justus-Liebig-University Giessen, Germany

## Abstract

**Background:**

Chronic hypoxia influences gene expression in the lung resulting in pulmonary hypertension and vascular remodelling. For specific investigation of the vascular compartment, laser-microdissection of intrapulmonary arteries was combined with array profiling.

**Methods and Results:**

Analysis was performed on mice subjected to 1, 7 and 21 days of hypoxia (FiO_2 _= 0.1) using nylon filters (1176 spots). Changes in the expression of 29, 38, and 42 genes were observed at day 1, 7, and 21, respectively. Genes were grouped into 5 different classes based on their time course of response. Gene regulation obtained by array analysis was confirmed by real-time PCR. Additionally, the expression of the growth mediators PDGF-B, TGF-β, TSP-1, SRF, FGF-2, TIE-2 receptor, and VEGF-R1 were determined by real-time PCR. At day 1, transcription modulators and ion-related proteins were predominantly regulated. However, at day 7 and 21 differential expression of matrix producing and degrading genes was observed, indicating ongoing structural alterations. Among the 21 genes upregulated at day 1, 15 genes were identified carrying potential hypoxia response elements (HREs) for hypoxia-induced transcription factors. Three differentially expressed genes (S100A4, CD36 and FKBP1a) were examined by immunohistochemistry confirming the regulation on protein level. While FKBP1a was restricted to the vessel adventitia, S100A4 and CD36 were localised in the vascular tunica media.

**Conclusion:**

Laser-microdissection and array profiling has revealed several new genes involved in lung vascular remodelling in response to hypoxia. Immunohistochemistry confirmed regulation of three proteins and specified their localisation in vascular smooth muscle cells and fibroblasts indicating involvement of different cells types in the remodelling process. The approach allows deeper insight into hypoxic regulatory pathways specifically in the vascular compartment of this complex organ.

## Background

Chronic pulmonary hypertension is associated with structural alterations of the large and small intrapulmonary arteries. Smooth muscle cells, endothelial cells and fibroblasts are involved in this process of vascular remodelling. A set of genes is known to be transcriptionally induced under hypoxic conditions by hypoxia-induced transcription factors (HIF) [1-4] and mice partially deficient for HIF-1α only develop attenuated pulmonary hypertension [5,6]. Several growth factors like PDGF (Platelet derived growth factor), FGF (Fibroblast growth factor) and TGF-β (Transforming growth factor-beta) have been shown to be induced during pulmonary vascular remodelling [7-9]. Finally, regulation of matrix-related genes like procollagens and MMPs (Matrix metalloproteinases) were also described to participate in this process [10,11]. However, a comprehensive set of genes involved in remodelling has not been identified and the time course of gene induction from the initial stimulus up to the structural changes is poorly understood.

Expression arrays can simultaneously determine regulation of a multitude of genes [12-14]. Applying arrays for analysis of hypoxia-induced gene regulation in the lung [13,14], the use of tissue homogenate results inevitably in an averaging of the various expression profiles of the different cell types. As intrapulmonary arteries represent only a minimal portion of the lung tissue (<10 %) the expression profile of this compartment may be largely masked or even lost when using lung homogenates. To overcome this problem, laser-microdissection techniques have been successfully employed and shown to precisely isolate single cells or compartments under optical control [15-17]. Recently, we subjected laser-microdissected intrapulmonary arteries to cDNA array profiling and showed that the expression signature of these isolated arteries differs remarkably from that of lung homogenates [18].

In this study we aimed to identify genes in the vascular compartment that are involved in the development of pulmonary hypertension and the process of lung vascular remodelling in response to hypoxia. Lungs from control mice and those exposed to normobaric hypoxia (FiO_2 _= 0.1) were excised and used to prepare tissue sections. After laser-microdissection of intrapulmonary arteries, extracted RNA was preamplified and subsequently hybridized to cDNA arrays. To determine the onset of expression changes among different genes and the time course of regulation, hypoxic time periods of 1, 7 and 21 days were selected. For validation of array-based differential gene expression, a subset of genes was independently measured by a combination of laser-microdissection and real-time PCR. Additionally, immunohistochemical analysis was performed for the three selected genes S100A4, CD36 and FKBP1a to determine protein regulation and localisation.

## Methods

### Lung preparation of mice under hypoxia/normoxia

Lungs were prepared as described previously [18]. All animal experiments were approved by the local authorities (Regierungspräsidium Giessen, no. II25.3-19c20-15(1) GI20/10-Nr.22/2000). In brief, male Balb/cAnNCrlBR mice (Charles River, Sulzfeld, Germany, 20–22 g) were exposed to normobaric hypoxia (inspiratory O_2 _fraction (FiO_2 _= 0.1)) in a ventilated chamber. Mice exposed to normobaric normoxia were kept in a similar chamber at a FiO_2 _of 0.21. After 1, 7 and 21 days, animals were intraperitoneally anesthetized (180 mg sodium pentobarbital/kg body weight), a midline sternotomy was performed, and the lungs were flushed via a catheter in the pulmonary artery (PA) with an equilibrated Krebs Henseleit buffer at room temperature. Afterwards, the airways were instilled with 800 μl prewarmed TissueTek^® ^(Sakura Finetek, Zoeterwoude, The Netherlands). After ligation of the trachea, the lungs were excised and immediately frozen in liquid nitrogen. Preparation of the hypoxic animals was continuously performed in the hypoxic environment.

### Laser-assisted microdissection

**Figure 1:**
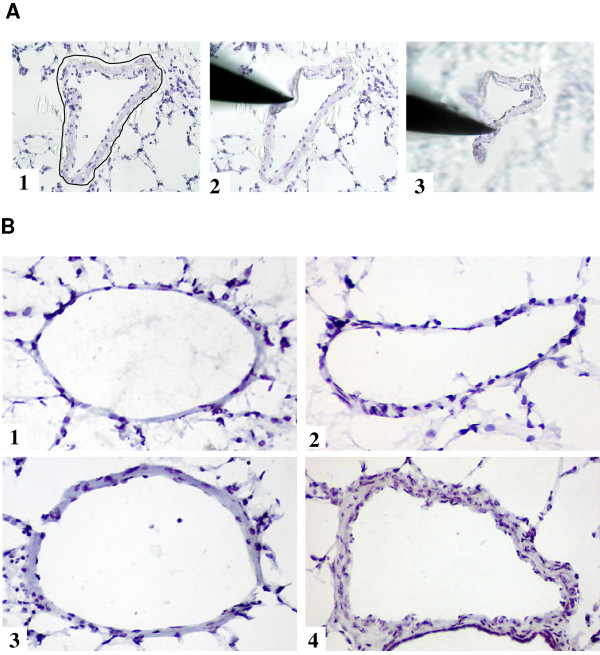
**Intrapulmonary arteries. **A) laser-microdissection of small intrapulmonary arteries. 1) The laser cuts along the outer side of the tunica adventitia. 2) A sterile needle is used to isolate the vessel. 3) Needle with adherent vessel is lifted and transferred afterwards to a reaction tube. Magnification × 200. B) Representative intrapulmonary arteries during the process of vascular remodelling. 1) Under normoxic conditions. 2) At day 1 of hypoxia. 3) At day 7 of hypoxia. Smooth muscle cell layer causes vascular thickening. 4) At day 21 of hypoxia. Magnification × 200.

Microdissection was performed as described in detail previously [18-20]. In brief, cryo-sections (10 μm) from lung tissue were mounted on glass slides. After hemalaun staining for 45 seconds, the sections were subsequently immersed in 70% and 96% ethanol and stored in 100% ethanol until use. No more than 10 sections were prepared at once to reduce the storage time. Intrapulmonary arteries with a diameter of 250–500 μm were selected and microdissected under optical control using the Laser Microbeam System (P.A.L.M., Bernried, Germany) (Figure 1A). Afterwards, the vessel profiles were isolated with a sterile 30 G needle. Needles with adherent vessels were transferred into a reaction tube containing 200 μl RNA lysis buffer.

### mRNA extraction

Messenger RNA isolation was performed according to the Chomczynski protocol with some modifications as previously described in detail [18]. After washing, RNA was resuspended in 10 μl RNase free H_2_O, and then subjected to DNase digestion (Ambion, Austin, TX; 1U, 30 min, 37°C). Afterwards, extraction was repeated and RNA was finally resuspended in 4 μl H_2_O.

### cDNA synthesis, amplification, labelling and hybridisation

These steps were performed as described previously [18]. Total RNA was reverse transcribed using the SMART™ PCR cDNA Synthesis Kit (Clontech, Palo Alto, CA). Complementary DNA was purified by the QIAquick™ PCR Purification Kit (Qiagen, Hilden, Germany) and eluted in 45 μl elution buffer (EB). From the eluted cDNA, 2 μl were separated for further determination of the amplification factor. For the PCR-based amplification, the remaining cDNA was mixed with 5 μl 10 × buffer, 1 μl PCR Primer (10 μM), 1 μl dNTP (10 mM) and 1 μl Advantage™ 2 Polymerase Mix. PCR conditions were 95°C for 1 min, followed by 19 cycles with 95°C for 15 s, 65°C for 30 s and 68°C for 3 min. The resulting PCR product was purified using the QIAquick™ columns as described above. Elution buffer (44 μl) was applied twice for elution and 2 μl were used to determine the amplification factor. All incubations were performed with a GeneAmp™ 2400 PCR cycler (PE Applied Biosystems, Foster City, USA).

The purified PCR product was labeled with α-^32^P dATP using the Atlas SMART™ Probe Amplification Kit (Clontech), purified by QIAquick™ columns, and eluted twice with 100 μl elution buffer. Hybridization was done at 68°C overnight on Mouse 1.2 II Atlas™ cDNA Arrays nylon filters with 1176 spotted cDNAs (Clontech). After washing, filters were exposed to an imaging plate (Fuji Photo Film, Tokyo, Japan). The plate was read with a phosphorimaging system (BAS RPI 1000, Fuji Photo Film).

### Analysis of array data

Raw data were collected using the AtlasImage™ 2.0 software (Clontech). Values of spot intensities were adjusted by a global normalization using the sum method provided by the software. The mean global background was calculated, and spots were considered to be present if the spot signal was at least two-fold higher than that.

For changes in transcript abundance, the normalized difference was used as a measure:



Here, I_N _is given by the adjusted intensity for the normoxia sample and I_H _by the adjusted intensity for the hypoxia sample, respectively.

For relatively small regulation (2–3 fold), D is comparable to the commonly used log-ratio of the intensities (log_2_(Q) with Q = I_H_/I_N_): D ≈ 0.5•log_2_(Q). The values of D have a codomain limited between -1 to +1: if either intensity equals 0, log(Q) cannot be determined meaningfully (log(Q) = ± ∞), whereas D gives -1 or +1 in these situations. Between -0.5 and +0.5 (2 fold regulation), both calculation methods give similar results.

The values can be transformed into each other by



The advantage of the normalized difference method over the log-ratio method is that genes with zero values (i.e., "on" and "off" regulation) can be included into further statistical analyses. Additionally, the variation of strongly regulated genes is decreased by expressing the changes as a difference instead of ratios.

In order to screen for relevant genes, the difference of the D values from zero was tested by a two-sided one-sample t-test. Those genes with p values ≤ 0.1 were considered to be potentially regulated genes as real-time PCR confirmed the regulation in >90%.

### Relative mRNA quantification by real-time PCR

To confirm the results obtained by nylon membrane hybridization, the regulation of a subset of genes was analyzed by real-time quantitative PCR using the ΔΔ C_T _method for the calculation of relative changes [21]. Real-time PCR was performed by the Sequence Detection System 7700 (PE Applied Biosystems). PBGD, an ubiquitously as well as consistently expressed gene that is free of pseudogenes was used as reference. For cDNA synthesis, reagents and incubation steps were applied as described previously (18). The reactions (final volume: 50 μl) were set up with the SYBR™Green PCR Core Reagents (Applied Biosystems) according to the manufacturer's protocol using 2 μl of cDNA. The oligonucleotide primer pairs are given in Table 1 (final concentration 200 nM). Cycling conditions were 95°C for 6 min, followed by 45 cycles of 95°C for 20 s, 58°C for 30 s and 73°C for 30 s. Due to the non-selective dsDNA binding of the SYBR™Green I dye, melting curve analysis and gel electrophoresis were performed to confirm the exclusive amplification of the expected PCR product.

**Table 1:** Primer sequences and amplicon sizes. The primer sets work under identical PCR cycling conditions to obtain simultaneous amplification in the same run. Sequences were taken from GeneBank, Accession numbers are given.

	**Genbank Accession**	**Primer Sequence (5' → 3')**		**Amplicon Length [bp]**
			
**Gene**		**Forward**	**Reverse**	
**PBGD**	M28664	GGTACAAGGCTTTCAGCATCGC	ATGTCCGGTAACGGCGGC	135
**Col1a1**	U08020	CCAAGGGTAACAGCGGTGAA	CCTCGTTTTCCTTCTTCTCCG	124
**Col1a2**	X58251	TGTTGGCCCATCTGGTAAAGA	CAGGGAATCCGATGTTGCC	113
**Col3 a1**	X52046	TCAAGTCTGGAGTGGGAGG	TCCAGGATGTCCAGAAGAACCA	92
**CA3**	M27796	GACGGGAGAAAGGCGAGTTC	CAGGCATGATGGGTCAAAGTG	101
**Mgp**	D00613	GTGGCGAGCTAAAGCCCAA	CGTAGCGCTCACACAGCTTG	101
**Myl6**	U04443	CTTTGAGCACTTCCTGCCCA	CCTTCCTTGTCAAACACACGAA	101
**Spi3**	U25844	TCCTGCCTCAAGTTCTATGAAGC	TGTTGATGTGCTGTCGGGAC	82
**Cytb245b**	M31775	TTTCGGCGCCTACTCTATCG	TCTGTCCACATCGCTCCATG	101
**Bzrp**	D21207	GAAACCCTCTTGGCATCCG	CCTCCCAGCTCTTTCCAGACT	105
**Psap**	U27340	GCAGTGCTGTGCAGAGATGTG	TCGCAAGGAAGGGATTTCG	104
**Tie2**	E08401	GCCGAAACATCCCTCACCT	TGGATCTTGGTGCTGGTTCAT	102
**PDGFb**	AF162784	CGCCTGCAAGTGTGAGACAAT	CGAATGGTCACCCGAGCTT	105
**SRF**	AB038376	GTCTCCCTCTCGTGACAGCAG	CAGTTGTGGGTACAGACGACGT	101
**VEGF-R1/FLT1**	D88689	GGAGCTTTCACCGAACTCCA	TCTCAGTCCAGGTGAACCGC	101
**TGF-β1**	M13177	GCCCTGGATACCAACTATTGCTT	AGTTGGCATGGTAGCCCTTG	127
**FGF2**	NM_008006	AGCGACCCACACGTCAAACT	CGTCCATCTTCCTTCATAGCAAG	104
**Tsp1**	J05605	ACAGTTGCACAGAGTGTCACTGC	CATTCACCATCAGGAACTGTGG	103
**CD36**	L23108	CCACTGCTTTCAAAAACTGGG	GCTGCTGTTCTTTGCCACG	101
**CD81**	X59047	CCTCAGGCGGCAACATACTC	GGCTGCAATTCCAATGAGGT	101
**FK506bp1a**	X60203	CAAGCAGGAGGTGATCCGAG	CGGTGGCTCCATAGGCATAG	104
**bFGF1 precursor**	X51893	TACAAGAAAACCACCAACGGC	CCAAAAGACCACACATCGCTC	101
**Il-9 receptor**	M84746	GGCAGCAGCGACTATTGCAT	ACACAGGAAGGGCCACAGG	115
**Cyt cVIIc**	X52940	GGTTCACGACCTCCGTGGT	CATCATAGCCAGCAACCGC	101
**Ogn**	D31951	GACCTGGAATCTGTGCCTCCT	ACGAGTGTCATTAGCCTTGCAG	114
**Ptbp1**	X52101	TGGTGTGGTCAAAGGCTTCA	GCAGTTCAATCAGCGCCTG	101
**S100A4**	D00208	AGGAGCTACTGACCAGGGAGCT	TCATTGTCCCTGTTGCTGTCC	103

### Hypoxia response element (HRE)

Genes regulated after 1 day of hypoxia treatment were screened for presence of hypoxia response elements (HRE). The consensus sequence chosen for HRE was "BACGTSSK", were B can be T, G or C; S – G or C and K – T or G. Regulated genes from 1 day array results were screened 5,000 bp downstream and upstream from coding sequence for the occurrence of this consensus sequence. Sequences were obtained from  (according to accession numbers given for the corresponding features on the nylon arrays).

### Biological processes

Accession numbers from genes being regulated in hypoxia conditions were subjected to screening biological processes by using Gene Ontology page, AmiGo: 

### Immunohistochemistry

Cryo-sections (10 μm thick) from lung tissue were mounted on Superfrost glass slides (R. Langenbrinck, Germany). Slides were dried overnight and stored at -20°C until use. Fixation was performed in acetone (Riedel-de Haen, Seelze) for 10 minutes. All antibodies were diluted in ChemMate™ Antibody Diluent, (Dako, Denmark). Following dilutions of primary antibodies were used: Rabbit polyclonal anti-human S100A4 antibody (Neomarkers, Fremont, CA) – 1:700, rabbit polyclonal anti-human FKBP1a antibody (Abcam, Cambridge, UK) – 1:300, rabbit polyclonal anti-human CD36 (Santa Cruz Biotech, California, USA) – 1:200. S100A4 and CD36 were incubated in a humid chamber overnight, while FK506BP (FKBP1a, FKBP12) was incubated for one hour. Afterwards, the slides were washed 3 × in TBS and incubated with the secondary antibody goat anti-rabbit IgG (Southern Biotech, Eching, Germany) – 1:150 for 40 min. After washing, alkaline phosphatase conjugated anti-goat antibody (Rockland, Gilbertsville, PA) – 1:200, 40 min was applied. Negative controls were performed with the omission of the first antibody.

## Results

### Animal model: Vascular remodelling

Prolonged exposure to hypoxia results in structural changes of small intrapulmonary arteries in mouse lungs. These changes are mainly characterised by thickening of media layer (proliferation of vascular smooth muscle cells) (Figure 1B).

### Array analysis

For each array analysis 30 to 40 vessel profiles (diameter 250–500 μm) were isolated from lung sections of animals kept in hypoxia (FiO_2 _0.1) and those kept in normoxia for 1, 7, and 21 days. In all cases, four independent hybridization experiments were performed. When comparing exposure to hypoxia against normoxia, 29 genes (19 up/10 down), 38 genes (18 up/20 down), and 42 genes (25 up/17 down) were regulated after 1, 7, and 21 days, respectively with a p-value ≤ 0.1 (Additional files 1, 2 and 3).

### Determination of regulation by real-time RT-PCR

**Figure 2:**
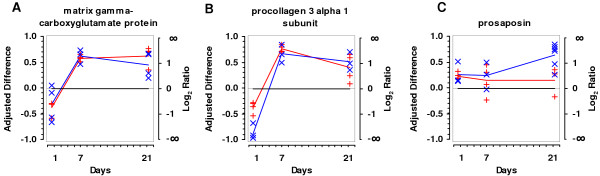
**Comparison of array based time course of expression to that obtained by real-time RT-PCR (red: array; blue: TaqMan). **A) Matrix γ-carboxyglutamate protein. B) Procollagen 3 α1. C) Prosaposin.

For all hypoxic time periods, subsets of genes were selected for independent determination of regulation by real-time RT-PCR using intrapulmonary arteries isolated by laser-microdissection. To confirm the array data, we randomly selected genes from the unified list of genes, but with a certain focus on genes with a regulation factor between 0.5 and 2. Three independent experiments were performed for each gene. Mean ± SEM is presented in the respective columns in additional files 1, 2 and 3. In total, 37 ratios of hypoxic to normoxic expression were determined. From these genes under investigation, 34 (95 %) were clearly confirmed to be up- or down-regulated. Only CD 81 failed to be ascertained at day 7. Although, most of the genes were regulated by less than factor 2 when assessed by array analysis, the vast majority of these regulations were confirmed by real-time PCR (Figure 2).

### Growth factor analysis

Among growth factors and receptors that were assumed to be regulated, sequences of PDGF (β-polypeptide), TGF-β1, TSP-2/TSP-1 (sequence homology 77%) and VEGF-R1 (Flt) were immobilized on the applied nylon filter. However, no hybridisation signal was detected for these genes. Therefore, relative mRNA levels of these genes together with FGF-2, Angiopoietin Receptor 2 (TIE2) and Serum Response Factor (SRF) were determined by real-time PCR from laser-microdissection from 1 and 7 days hypoxic/normoxic intrapulmonary arteries (Table 2). All transcripts were detected by real-time RT-PCR. PDGF-B and TSP-1 showed an upregulation after 1 and 7 days of hypoxia, TIE-2, TGF-β and SRF only after 7 days. VEGF-R1 mRNA was increased after 1 day, but decreased after 7 days. FGF-2 was slightly downregulated in hypoxia.

**Table 2:** Growth factors determined by real-time PCR. Among growth factors and receptors that were described to be regulated, TSP-1, VEGF-R1 (Flt), PDGF-B, Serum Response Factor (SRF), TGF-β 1, Angiopoietin Receptor 2 (TIE2) and FGF-2 were separately determined by relative mRNA quantification after laser-microdissection from 1 and 7 day hypoxic/normoxic intrapulmonary arteries. Mean ± SEM is given from n = 4 independent experiments.

**Genes**	**1 Day Hypoxia**	**7 Days Hypoxia**
Thrombospondin 1 (TSP-1)	4.61 ± 0.79	1.95 ± 0.44
VEGF-R1/FLT1	2.38 ± 0.43	0.61 ± 0.13
PDGF-β	1.41 ± 0.28	2.96 ± 0.82
Serum Response Factor (SRF)	1.09 ± 0.08	1.70 ± 0.29
Transforming Growth Factor β 1 (TGF-β 1)	0.94 ± 0.14	2.10 ± 0.46
Angiopoietin Receptor 2 (TIE2)	0.91 ± 0.09	1.94 ± 0.21
Fibroblast Growth Factor 2 (FGF-2)	0.75 ± 0.14	0.80 ± 0.15

### Classification of genes according to biological processes

Genes were grouped in nine classes according to their biological processes:

Organogenesis (angiogenesis, muscle development), cell adhesion/cell organisation, signal transduction, cell growth and/or maintenance (cell cycle, lipid transport, ion transport), immune response (antigen presentation, immune cell activation), proteolysis and peptidolysis, transcription/translation process (DNA packaging and repair, RNA processing, protein biosynthesis), energy metabolism/electron transport (carbohydrate metabolism, lipid catabolism, electron transport, removal of superoxide radicals), unknown (biological processes not known for mouse or human genes).

**Figure 3:**
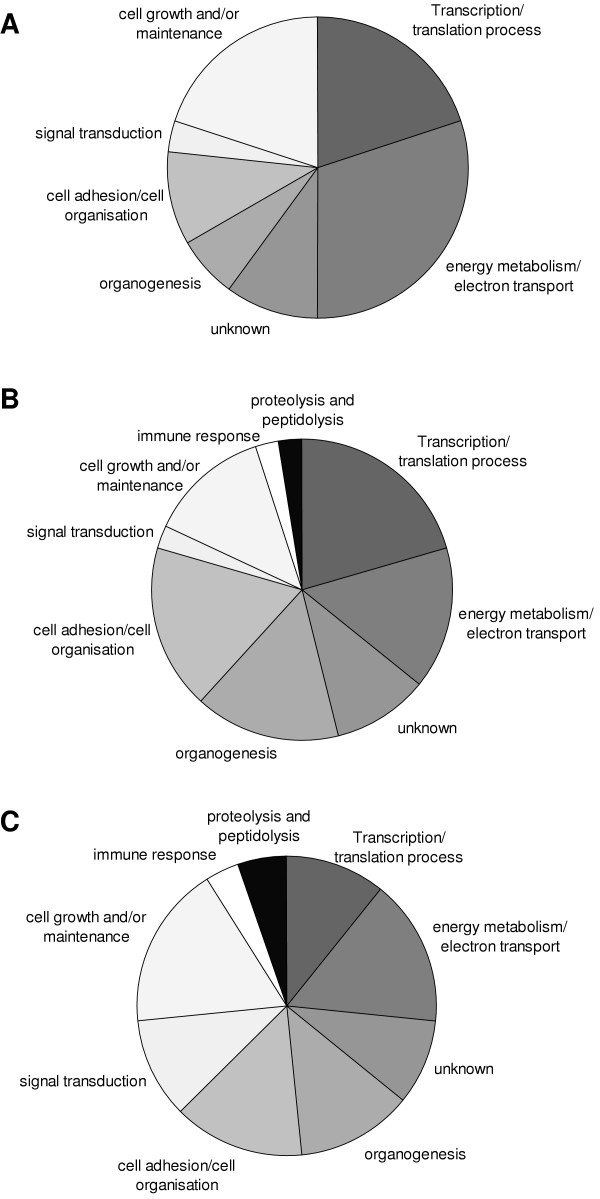
**Gene classification according to biological processes. **Significantly regulated genes were grouped according to their biological processes from NCBI, Gene Ontology, AmiGo. A) 1 day hypoxia, B) 7days hypoxia, C) 21days hypoxia.

The sizes of the pie charts in Figure 3 correspond to the contribution of genes involved in one of the biological processes. After 1 day of hypoxia most regulated genes (> 35%) responsible for metabolism, while at later time points this group was less prominent (~20% for 7 and 21 days). With continued exposure to hypoxia the subset of regulated genes responsible for organogenesis (3.5%, 13%, and 9% for 1, 7 and 21 days, respectively) and immune response (0%, 3%, and 7% for 1, 7 and 21 days respectively) was increased.

### Genes potentially regulated by hypoxia-inducible transcription factor (HIF) responsive element (HRE)

**Figure 4:**
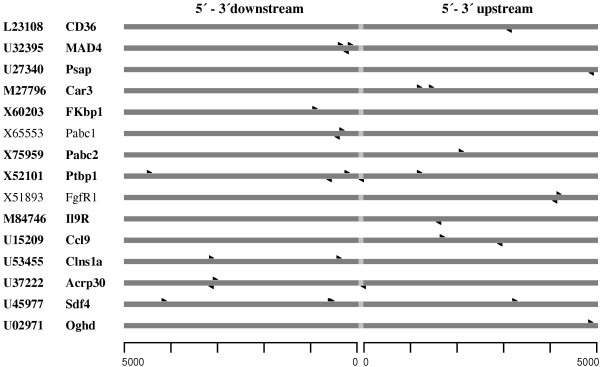
**Putative HIF-responsive elements (HRE) of the genes upregulated at day 1. **Twenty genes were screened for the presence of the consensus sequence "BACGTSSK" 5000 bp up- and downstream the coding sequence. Aldolase C, a known HIF-responsive gene, was excluded. Fifteen genes were found carrying one or more putative HREs.

The genomic context of genes upregulated after 1 day was screened 5,000 bp downstream and upstream from coding sequence for the presence of the HIF-responsive element consensus sequence "BACGTSSK". Among those genes some were carrying HRE (e.g. CD36, and MAD4), while others did not have any (e.g. apolipoprotein D). From 17 different possible variants of HRE, four: CACGTGGT, GACGTGGG, CACGTGCT and TACGTGGG were found to be the most common sequences (47% of all HRE) see Figure 4.

### Regulation and protein localisation of CD36, S100A4, and FKBP1a

**Figure 5:**
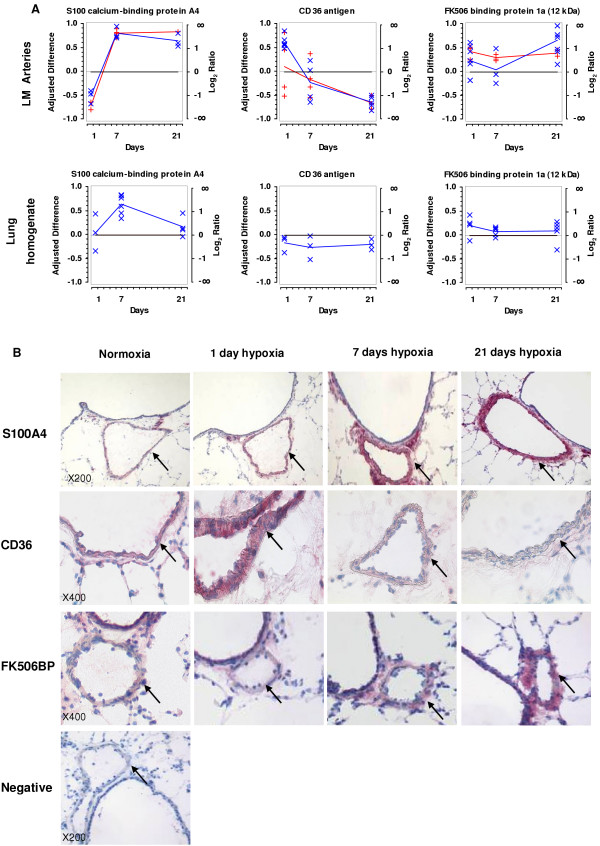
**Regulation of S100A4, CD36 and FKBP1a on mRNA and protein level. **A) Comparison of regulation between laser-microdissected arteries and lung homogenate from 1, 7, and 21 days of hypoxia exposure. (Red: array; blue: TaqMan). B) Immunohistochemical staining of S100A4, CD36 and FKBP1a in the mouse lung.

Three genes (CD36, S100A4, and FKBP1a) were selected for further characterisation. From the array data, CD36 showed a mean of 1.1 at day 1 and 0.9 at day 7 (both unregulated), with a remarkable standard deviation. Using real-time RT-PCR, upregulation (2.9 ± 0.56) was observed at day 1 and a slight downregulation at day 7, but also with high deviation (0.7 ± 0.29) (Figure 5A and additional files 2 and 3). On the other hand, the data from the arrays and real-time RT-PCR for S100A4 and FKBP1a showed strong correlation in upregulation during prolonged hypoxia exposure.

We also examined whether the expression levels of CD36, S100A4, and FKBP1a could have been detected by real-time RT-PCR using lung homogenate. Interestingly, only S100A4 was significantly regulated at day 7 of hypoxia exposure, while no regulation was observed for any of the other genes at all time points (Figure 5A).

**Figure 6:**
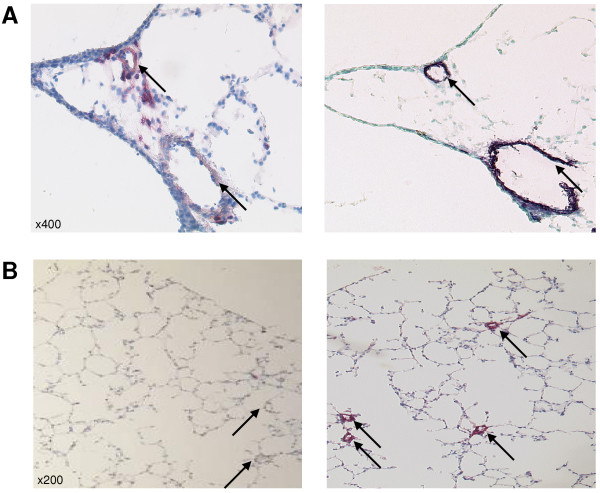
**Immunolocalisation of S100A4. **A) S100A4 protein (left panel) co-localises with alpha-smooth muscle actin (right panel). B) Small vessels (marked by arrows) are negative for S100A4 under normoxia (left panel) however stain positive for S100A4 after 21 days of hypoxia.

Regulation was then investigated on the protein level by immunohistochemistry (Figure 5B). CD36, S100A4, and FKBP1a showed a similar time course of protein expression as predicted by real-time RT-PCR. S100A4 and CD36 were localised exclusively to smooth muscle cells, whilst FKBP1a expression was restricted to the adventitia. Localisation of S100A4 was confirmed by the co-localisation with anti-alpha smooth muscle actin on serial sections (Figure 6A). After prolonged hypoxic exposure (7 and 21 days) S100A4 was additionally located in neo-muscularised resistance vessels (Figure 6B).

## Discussion

cDNA arrays have been shown to be powerful tools for the broad analysis of the transcriptome. The combination with laser-microdissection reveals compartment- or even cell-type specific gene regulation within complex tissues and organs [22-24] that may be masked using tissue homogenate (Figure 5a). Indeed, when comparing tissue homogenates to intrapulmonary arteries, the whole expression profiles differed completely [18]. Thus, the presented study is focusing on microdissected intrapulmonary arteries for the analysis of gene expression underlying hypoxic vascular remodelling.

### Technical aspects

#### Statistical analysis

For measurement of differential gene expression, the ratio of intensities is usually calculated after normalization. For genes with intensity values close to background or even absent in one condition, the ratio cannot be calculated. Consequently, these genes are excluded from statistical analysis although they are obviously regulated. To overcome this problem, the differences of the background-corrected and normalized intensities were used instead of their ratios. However, among the genes measured independently by real-time PCR, 95% were confirmed in regulation (e.g. osteoglycin after 1 d, cytochrome b-245 alpha polypeptide after 21 d).

#### Technical limitations

A couple of reasons may cause a discrepancy of the results obtained from arrays and real-time PCR:

Filter-based micro arrays have a limited dynamic range. This mainly is due to the fact that images have to be acquired where the intensity information is coded into 16-bit variables [25,26]. Real-time PCR offers a significantly higher dynamic range for detection that is more than 20,000-fold higher than the range of arrays obtained from 16-bit images [27,28]. Additionally, cross-hybridisation on the arrays may reduce the dynamic range or even completely cover differences, especially of low abundant genes [29]. Furthermore, micro arrays with several hundreds or even several thousands of sequences are hybridised at one temperature. As the immobilized sequences may vary a bit in their optimum hybridisation temperature, some labelled products may show suboptimal hybridisation efficiencies at the given temperature. Finally, low-abundant transcripts may not yield enough signal and fail to be detected by array analysis but are easily identified by quantitative RT-PCR. Consequently, both sensitivity and precision limit the ability to detect and identify regulated genes by arrays. Due to these limitations coupled with statistical restrictions, array data should be confirmed by real-time PCR. Following this line, some important genes (i.e., VEGF-R1, TGF-β) known to be involved in the remodelling process [7,30,31] were expected to be regulated in response to hypoxia. As these genes failed to be positive by array analysis, we performed real-time RT-PCR. By this more sensitive technique, the genes were detected throughout and regulation levels could be determined. We conclude that the absence of labelled spots does not necessarily indicate the absence of the gene's mRNA.

Furthermore, utilising nylon filters with 1176 spotted genes some gene subsets were absent, including several interesting candidates in hypoxia induced regulation, e.g., ion channels, some growth and transcription factors. With potential importance for our focus of the remodelling process, we exemplarily analysed some additional genes by real-time PCR (FGF-2, TIE2, Serum Response Factor).

### Differential gene expression and time courses

Among the genes with potential regulation, some showed differential expression at one, two or all three different time points. While some genes have already been mentioned to be involved in hypoxia-induced vascular remodelling (e.g. procollagens; [10], many others are shown to be related to this process for the first time. As expected, hypoxia did not turn out to be a dramatic stimulus for expression changes, and only few genes were measured to be upregulated with more than factor two (i.e., procollagens after 7 and 21 days), or to be downregulated to the same extent (i.e., CD36 after 21 days). After 1 day of hypoxia, ion-binding genes (45-kDa calcium-binding protein precursor, S100 calcium binding protein A4, chloride ion current inducer protein) as well as transcription modulating genes (MAD4, poly A binding proteins, and polypyrimidine tract binding protein) were predominantly regulated. FK506 binding protein 1a is well known to be involved in cell cycle regulation [32], but also in contraction-associated Ca^2+ ^release from the sarcoplasmatic reticulum [33]. This may indicate altered ion homeostasis in response to hypoxia as well as transcriptional preparation and initiation of long-term modifications in the vascular cells. Growth stimulus via increased expression of VEGF-R1, TSP-1, and PDGF fits well into this view. Interleukin 9 receptor, a T(H)2-type cytokine receptor, showed increased expression after 1 day, followed by downregulation after 21 days. Interestingly, it was also found to be upregulated in fibroblasts derived from an aortic aneurysm [34]. After 7 days, PDGF and TSP-1 were still increased as compared to controls. Serum responsive factor (SRF), angiopoietin 2 receptor (TIE2), fibroblast inducible secreted protein (FISP, mouse homolog of mda-7/Il-24) and TGF-β joined the upregulated growth and angiopoesis mediators. The production of matrix was apparently increased, as indicated by enhanced expression of fibronectin, matrix gamma carboxyglutamate protein and procollagen subunits. Vasodilator-stimulated phosphoprotein (VASP), a substrate of NO targeted cGMP dependent protein kinase [35] that is involved in fibroblast migration [36] was also upregulated. After 21 days, while the matrix production was still ongoing, reconstruction by proteases (carboxypeptidase E, serine proteinase inhibitor 2.2) additionally occurred.

**Figure 7:**
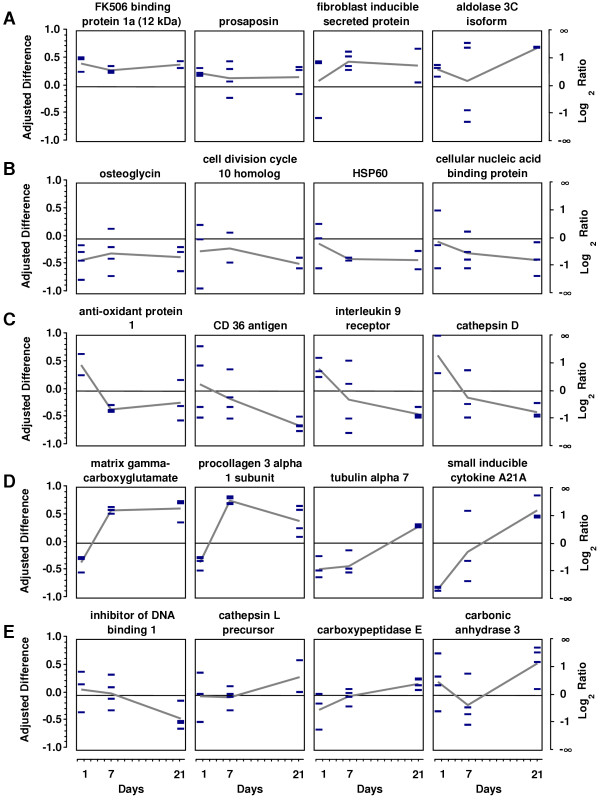
**Classification of genes with similar regulation pattern. Four representatives each are given. **A) Continuous upregulation at day 1, 7, and 21. B) Continuous downregulation at day 1, 7, and 21. C) Primarily upregulated, afterwards decrease under normoxic level (= downregulation). D) Primarily downregulated, afterwards increase over normoxic level (= upregulation). E) Primarily not regulated, afterwards up- or downregulated ("late response").

To identify possible regulation mechanisms, we defined groups of genes exhibiting similar time courses of differential gene expression. Examples of these groups are given in Figure 7. First, we grouped genes that were upregulated throughout all time points. Representatives are FK506 binding protein 1a (12 kDa), prosaposin, fibroblast inducible secreted protein (FISP) and aldolase 3C isoform. In contrast, we found genes that were downregulated throughout (i.e., osteoglycin, cell division cycle 10 homolog, HSP 60, cellular nucleic acid binding protein). Furthermore, some genes were upregulated after 1 day, but strongly decreased afterwards, dropping below the normoxic level (i.e., anti-oxidant protein 1, CD36, interleukin 9 receptor, cathepsin D). Another group showed initial downregulation, but increased afterwards above the normoxic level (i.e., matrix gamma carboxyglutamate protein, procollagen 3α 1 subunit, tubulin alpha 7, small inducible cytokine A21A). Finally, some genes seem to be unregulated at early stages, but were at later stages up- or downregulated ("late response"). Genes belonging to this group are inhibitor of DNA binding 1, cathepsin L precursor, carboxypeptidase E and carbonic anhydrase 3.

Even if some of these data vary and may lead to slight changes in the classification of the genes, fairly consistent profiles were noted for many genes. In addition, many time-courses were confirmed by real-time PCR-derived measurements (see Additional files 1, 2, and 3). When directly comparing the array-based regulation profile to that based on real-time PCR (Figure 2 and 5A), excellent correlation was found for matrix gamma-carboxyglutamate protein, procollagen 3α1 subunit, S100 calcium binding protein A4 and FK506 binding protein 1a. The level of prosaposin upregulation when measured by real-time PCR was greater than by arrays at day 21. CD36 varied considerably at day 1 and 7 using both techniques. While array measurements did not allow allocation of this gene definitely to group C or E, relative mRNA quantification indicated primary upregulation and thus inclusion to group C. Overall, the possibility to allocate many genes to one of these five groups supports the hypothesis that these genes may be regulated by common mechanisms and regulatory elements, although not being primarily related.

Most of the genes regulated in array experiments were responsible for metabolism. Hypoxia regulates many genes involved in glycolysis [37-39], lipid pathways [40,41], protein synthesis and degradation [42,43]. The expression of metabolic genes was more pronounced at the early time point (1 day of hypoxia), which might indicate an adaptative response. Moreover, with increased duration of hypoxia more genes responsible for angiogenesis were upregulated. This finding matches perfectly to reports, which demonstrate vascular remodelling after prolonged exposure to hypoxia [44-46].

Due to the potential discrepancy between mRNA and the protein levels, we applied immunohistochemical staining to analyse protein expression. All three investigated proteins (S100A4, CD36 and FKBP1a), showed good correlation to mRNA expression levels. S100A4 and CD36 were localised exclusively to smooth muscle cells, while FKBP1a expression was restricted to the adventitia (Figure 5B). At later time points (7 and 21 days), we additionally found S100A4 in newly muscularized small vessels. Interestingly, approximately 5% of mice overexpressing S100A4 develop spontaneously pulmonary arterial lesions similar to that seen in patients with pulmonary vascular disease [47]. Lawire *et al. *have recently described that induction of S100A4 by serotonin induces migration of human pulmonary artery SMC [48]. In accordance with these studies, the observed upregulation of S100A4 and localisation to small vessels indicates an ongoing remodelling process stimulated by hypoxia. CD36 has been associated with many processes such as scavenger receptor functions, lipid metabolism, fatty acid transport, angiogenesis, cardiomyopathy and TGF-β activation [49]. Therefore, its higher expression in arteries after 1 day hypoxia exposure may indicate adaptation to low oxygen tension. Another protein, FKBP1a was more abundant in later hypoxia time points and was already shown to be involved in cell cycle regulation and Ca^2+ ^homeostasis [32,33]. Moreover, FKBP1a was found to be activated via ERK-R and AKT pathway leading to the HIF-2α nuclear translocation and subsequent transcription of target genes responsible for increased angiogenesis and proliferation [50].

### Genes potentially regulated by hypoxia-inducible transcription factors (HIF)

Alveolar hypoxia leads to vasoconsrtiction of pulmonary arteries. Chronic hypoxia downregulates expression of voltage-gated potassium channels [51], resulting in depolarisation of smooth muscle cells, subsequent Ca^2+ ^influx and increased vasoconstriction. Small intrapulmonary vessels appear to react stronger to oxygen deprivation than larger vessels. This might be due to different expression level of potassium channels on both types of vessels. Supporting this hypothesis, Archer *et al. *have shown preferential expression of voltage-gated potassium channels in resistance pulmonary arteries [52].

In addition to increased cytoplasmic Ca^2+ ^levels, another important effectors for hypoxic remodelling are hypoxia-inducible transcription factors (HIF) [1-3]. The binding to HIF-responsive elements (HREs) following nuclear translocation results in an increased transcription of the respective genes. Both, the HIF-1α and HIF-2α subunits undergo hypoxia-induced protein stabilisation and bind identical target DNA sequences [53]. After defining a consensus sequence for the HREs [54], several dozen genes have been revealed to possess HREs [3,4]. Moreover, using reporter assays regulation was confirmed to be HIF dependant (i.e., erythropoietin; ref. [55]). Among the genes positively detected on the nylon filters, aldolase C is known to be regulated in a HIF-dependent manner [4] and was upregulated at all time points (Figure 7, group A). Glyceraldehyde-3-phosphate dehydrogenase (GAPDH), another HRE-carrying gene, was found to be upregulated at day 7 and 21. However, in arrays from day 1 the GAPDH spot intensity was maximum for both normoxia and hypoxia, and a ratio could not be calculated. We investigated the genes upregulated at 1 day (Additional file 1) for the presence of HRE. From the 21 upregulated genes identified by array analysis, we screened 5000 bp up- and downstream of the coding sequence for the presence of the consensus sequence "BACGTSSK" [54]. Putative HREs were detected in 15 genes (Figure 4). Interestingly, 4 from 17 possible sequence variants that had the highest occurrence were also found in well-known HIF-1 regulated genes (VEGF, EPO, ENO1, and GAPDH). This finding underlines the importance of genes carrying the above mentioned sequences. Respective genes may be HIF-induced, which remains to be confirmed in the future by reporter gene assays or electrophoretic mobility shift analysis. On the other hand, in six upregulated genes no HRE consensus sequences could be found. These genes may be induced by a HIF dependent hypoxia-responsive element not represented by the above given consensus sequence. Alternatively, these genes may be indirectly regulated by another, primarily HIF-induced gene. Additionally, other regulatory pathways may exist to upregulate genes in a hypoxia dependent manner.

## Conclusion

Combining laser-microdissection and cDNA array analysis allows a compartment-specific broad gene expression analysis of intrapulmonary arteries in a model of hypoxia-induced pulmonary hypertension. Sets of genes were found to be up- or downregulated at 1, 7 and 21 days of hypoxia reflecting different states of vascular remodelling. According to similar time courses of differential expression, 5 groups were classified indicating common regulation mechanisms. Among the genes upregulated at day 1, several carry putative HIF responsive transcription elements while others do not. This may suggest alternative pathways of hypoxia sensing and downstream gene regulation. Immunohistochemistry confirmed regulation of three proteins and specified their localisation in vascular smooth muscle cells (S100A4, CD36) and fibroblasts (FKBP1a) indicating involvement of the different cells types in the remodelling process. Thus, our approach revealed several new genes involved in the process of hypoxic lung vascular remodelling and allows deeper insight into the underlying mechanisms of the vascular lung compartment.

## Authors' contributions

GK: laser-microdissection, arrays, real-time PCR, immunohistochemistry, preparation of the manuscript

JW: analysis of array data and real-time PCR data

SW: laser-microdissection, arrays, real-time PCR

IL: immunohistochemistry, real-time PCR

IRK: advice and discussion of statistical calculation

AZ: advice and discussion of statistical calculation

WS: design of project, discussion of data

RMB: introduction to laser-microdissection, analysis of immunohistochemistry and histopathology

NW: animal model of hypoxia induced pulmonary hypertension, discussion of data

LF: coordination and design of project, preparation of the manuscript

All authors have read and approved the finial manuscript.

## Supplementary Material

Additional File 1 **List of genes up- or down-regulated at day 1 of hypoxia**. For changes in transcript abundance, the normalized difference D was used as a measure (see Methods). The D derived Q(D) is given and compared to the commonly used ratio of the intensities Q = I_H_/I_N. _If either intensity equals 0, log_2_(Q) cannot be determined meaningfully, whereas D gives -1 or +1 in these situations. This allows to include genes with zero values (i.e., "on" and "off" regulation) into further statistical analyses. In order to screen for relevant genes, the difference from zero of the D values was tested by a two-sided one-sample t-test. Those genes with p-values ≤ 0.1 were considered to be potentially regulated as real-time PCR confirmed in >90% the regulation. TaqMan PCR derived ratios are given as mean ± standard error of mean (SEM). Click here for file

Additional File 2 **List of genes up- or down-regulated at day 7 of hypoxia**. For changes in transcript abundance, the normalized difference D was used as a measure (see Methods). The D derived Q(D) is given and compared to the commonly used ratio of the intensities Q = I_H_/I_N. _If either intensity equals 0, log_2_(Q) cannot be determined meaningfully, whereas D gives -1 or +1 in these situations. This allows to include genes with zero values (i.e., "on" and "off" regulation) into further statistical analyses. In order to screen for relevant genes, the difference from zero of the D values was tested by a two-sided one-sample t-test. Those genes with p-values ≤ 0.1 were considered to be potentially regulated as real-time PCR confirmed in >90% the regulation. TaqMan PCR derived ratios are given as mean ± standard error of mean (SEM). Click here for file

Additional File 3 **List of genes up- or down-regulated at day 21 of hypoxia**. For changes in transcript abundance, the normalized difference D was used as a measure (see Methods). The D derived Q(D) is given and compared to the commonly used ratio of the intensities Q = I_H_/I_N. _If either intensity equals 0, log_2_(Q) cannot be determined meaningfully, whereas D gives -1 or +1 in these situations. This allows to include genes with zero values (i.e., "on" and "off" regulation) into further statistical analyses. In order to screen for relevant genes, the difference from zero of the D values was tested by a two-sided one-sample t-test. Those genes with p-values ≤ 0.1 were considered to be potentially regulated as real-time PCR confirmed in >90% the regulation. TaqMan PCR derived ratios are given as mean ± standard error of mean (SEM). Click here for file
