# Supplementary material for: Expression profiling of laser-microdissected intrapulmonary arteries in hypoxia-induced pulmonary hypertension
Source: Respir Res. 2005 Sep 19;6(1):109. doi: 10.1186/1465-9921-6-109 (PMC1261535; doi:10.1186/1465-9921-6-109)
Supplement: Additional File 1 — List of genes up- or down-regulated at day 1 of hypoxia. For changes in transcript abundance, the normalized difference D was used as a measure (see Methods). The D derived Q(D) is given and compared to the commonly used ratio of the intensities Q = IH/IN. If either intensity equals 0, log2(Q) cannot be determined meaningfully, whereas D gives -1 or +1 in these situations. This allows to include genes with zero values (i.e., "on" and "off" regulation) into further statistical analyses. In order to screen for relevant genes, the difference from zero of the D values was tested by a two-sided one-sample t-test. Those genes with p-values ≤ 0.1 were considered to be potentially regulated as real-time PCR confirmed in >90% the regulation. TaqMan PCR derived ratios are given as mean ± standard error of mean (SEM). [file 1465-9921-6-109-S1.doc]

| **Gen** | **Genbank** |  | **Adjusted Difference: D** | | |  | **Adjusted Ratio: Q** | |  | **TaqMan: Q** |
| --- | --- | --- | --- | --- | --- | --- | --- | --- | --- | --- |
|  | **ID** |  | **Mean** | **Q(D)** | **p** |  | **Mean** | **p** |  | **Mean±sem** |
| small inducible cytokine A9 | U15209 |  | **0.86** | **7.1** | 0.008 |  | **4.8** | 0.093 |  |  |
| poly A binding protein cytoplasmic 2 | X75959 |  | **0.73** | **3.7** | 0.015 |  | **3.7** | 0.032 |  |  |
| lipocalin 2 | X14607 |  | **0.73** | **3.7** | 0.029 |  | **7.8** | 0.269 |  |  |
| max dimerization protein 4 (MAD4) | U32395 |  | **0.72** | **3.5** | 0.008 |  | **3.5** | 0.017 |  |  |
| 45-kDa calcium-binding protein precursor (CAB45); stromal cell-derived factor 4 (SDF4) | U45977 |  | **0.66** | **2.9** | 0.002 |  | **2.9** | 0.005 |  |  |
| carbonic anhydrase 1 | M32452 |  | **0.66** | **2.9** | 0.045 |  | **2.9** | 0.080 |  |  |
| adipocyte complement related protein of 30 kDa | U37222 |  | **0.60** | **2.5** | 0.063 |  | **2.5** | 0.102 |  |  |
| apolipoprotein D | L39123 |  | **0.58** | **2.4** | 0.037 |  | **2.6** | 0.103 |  |  |
| dihydrolipoamide dehydrogenase | U73445 |  | **0.56** | **2.3** | 0.077 |  | **2.3** | 0.119 |  |  |
| chloride ion current inducer protein (CLCI) | U53455 |  | **0.49** | **2.0** | 0.015 |  | **2.0** | 0.029 |  |  |
| basic fibroblast growth factor receptor 1 precursor (BFGF-R; FGFR1); FLG | X51893 |  | **0.48** | **1.9** | 0.021 |  | **1.9** | 0.039 |  | **2.3±0.8** |
| oxoglutarate dehydrogenase (lipoamide) | U02971 |  | **0.45** | **1.8** | 0.092 |  | **2.0** | 0.146 |  |  |
| poly A binding protein cytoplasmic 1 | X65553 |  | **0.42** | **1.7** | 0.020 |  | **1.7** | 0.031 |  |  |
| FK506 binding protein 1a (12 kDa) | X60203 |  | **0.42** | **1.7** | 0.037 |  | **1.7** | 0.053 |  | **1.9±0.3** |
| polypyrimidine tract binding protein | X52101 |  | **0.41** | **1.7** | 0.070 |  | **1.8** | 0.099 |  | **3.0±0.9** |
| interleukin 9 receptor | M84746 |  | **0.39** | **1.6** | 0.066 |  | **1.7** | 0.106 |  |  |
| pyruvate dehydrogenase E1alpha subunit | M76727 |  | **0.33** | **1.5** | 0.021 |  | **1.5** | 0.038 |  |  |
| aldolase C isoform | S72537 |  | **0.30** | **1.4** | 0.006 |  | **1.4** | 0.009 |  |  |
| prosaposin | U27340 |  | **0.23** | **1.3** | 0.005 |  | **1.3** | 0.008 |  | **1.5±0.3** |
| procollagen 3 alpha 1 subunit | X52046 |  | **-0.35** | **0.7** | 0.009 |  | **0.6** | 0.019 |  | **0.2±0.1** |
| matrix gamma-carboxyglutamate protein | D00613 |  | **-0.35** | **0.6** | 0.012 |  | **0.6** | 0.028 |  | **0.7±0.2** |
| osteoglycin | D31951 |  | **-0.38** | **0.6** | 0.067 |  | **0.6** | 0.132 |  | **0.6±0.3** |
| aspartylglucosaminidase | S81393 |  | **-0.39** | **0.6** | 0.085 |  | **0.5** | 0.156 |  |  |
| soluble superoxide dismutase 1 | M35725 |  | **-0.43** | **0.6** | 0.060 |  | **0.6** | 0.080 |  |  |
| tubulin alpha 7 | M13443 |  | **-0.48** | **0.5** | 0.009 |  | **0.5** | 0.019 |  |  |
| surfactant associated protein A | S48768 |  | **-0.59** | **0.4** | 0.030 |  | **0.3** | 0.050 |  |  |
| S100 calcium-binding protein A4 | D00208 |  | **-0.69** | **0.3** | 0.069 |  | **0.3** | 0.129 |  | **0.5±0.1** |
| small inducible cytokine A21A precursor | U88322 |  | **-0.84** | **0.2** | <0.001 |  | **0.2** | 0.006 |  |  |
| keratinocyte lipid binding protein | X70100 |  | **-0.99** | **0.0** | 0.005 |  | **0.0** | n.d. |  |  |
| carbonic anhydrase 3 | M27796 |  | **0.23** | **1.3** | 0.364 |  | **1.5** | 0.356 |  | **3.0±1.1** |
| CD 36 antigen | L23108 |  | **0.11** | **1.1** | 0.741 |  | **1.3** | 0.620 |  | **2.9±0.6** |
