# Supplementary material for: Expression profiling of laser-microdissected intrapulmonary arteries in hypoxia-induced pulmonary hypertension
Source: Respir Res. 2005 Sep 19;6(1):109. doi: 10.1186/1465-9921-6-109 (PMC1261535; doi:10.1186/1465-9921-6-109)
Supplement: Additional File 2 — List of genes up- or down-regulated at day 7 of hypoxia. For changes in transcript abundance, the normalized difference D was used as a measure (see Methods). The D derived Q(D) is given and compared to the commonly used ratio of the intensities Q = IH/IN. If either intensity equals 0, log2(Q) cannot be determined meaningfully, whereas D gives -1 or +1 in these situations. This allows to include genes with zero values (i.e., "on" and "off" regulation) into further statistical analyses. In order to screen for relevant genes, the difference from zero of the D values was tested by a two-sided one-sample t-test. Those genes with p-values ≤ 0.1 were considered to be potentially regulated as real-time PCR confirmed in >90% the regulation. TaqMan PCR derived ratios are given as mean ± standard error of mean (SEM). [file 1465-9921-6-109-S2.doc]

| **Gen** | **Genbank** |  | **Adjusted Difference: D** | | | | |  | **Adjusted Ratio: Q** | | |  | **TaqMan: Q** |
| --- | --- | --- | --- | --- | --- | --- | --- | --- | --- | --- | --- | --- | --- |
|  | **ID** |  | **Mean** | | **Q(D)** | **p** | |  | **Mean** | | **p** |  | **Mean±sem** |
| procollagen 1 alpha 1 subunit | U08020 |  | **0.83** | **6.0** | | | <0.001 |  | **6.5** | 0.004 | |  | **14.9±3.5** |
| S100 calcium-binding protein A4 | D00208 |  | **0.79** | **4.7** | | | <0.001 |  | **4.9** | 0.003 | |  | **6.4±3.0** |
| procollagen 1 alpha 2 subunit (COL1A2) | X58251 |  | **0.77** | **4.4** | | | <0.001 |  | **4.5** | <0.001 | |  | **5.8±1.9** |
| procollagen 5 alpha 2 subunit (COL5A2) | L02918 |  | **0.76** | **4.2** | | | 0.001 |  | **4.7** | 0.014 | |  |  |
| procollagen 3 alpha 1 subunit | X52046 |  | **0.75** | **4.1** | | | <0.001 |  | **4.2** | 0.002 | |  | **3.5±1.1** |
| testis cytochrome c | X55771 |  | **0.62** | **2.6** | | | 0.051 |  | **2.7** | 0.085 | |  |  |
| fibronectin 1 | X93167 |  | **0.58** | **2.4** | | | 0.010 |  | **2.6** | 0.032 | |  |  |
| matrix gamma-carboxyglutamate protein | D00613 |  | **0.58** | **2.4** | | | <0.001 |  | **2.4** | 0.001 | |  | **2.8±0.5** |
| histidine triad nucleotide-binding protein (HINT); protein kinase C iota (PKCI) | U60001 |  | **0.49** | **2.0** | | | 0.007 |  | **2.0** | 0.015 | |  |  |
| fibroblast inducible secreted protein | M70642 |  | **0.45** | **1.8** | | | 0.011 |  | **1.9** | 0.024 | |  |  |
| CD 63 antigen | D16432 |  | **0.44** | **1.8** | | | 0.007 |  | **1.8** | 0.020 | |  |  |
| cytoplasmic beta-actin (ACTB) | M12481 |  | **0.39** | **1.6** | | | 0.056 |  | **1.7** | 0.082 | |  |  |
| vasodilator-stimulated phosphoprotein | AF084548 |  | **0.35** | **1.5** | | | 0.073 |  | **1.6** | 0.114 | |  |  |
| FK506 binding protein 1a (12 kDa) | X60203 |  | **0.29** | **1.4** | | | 0.016 |  | **1.4** | 0.024 | |  | **1.0(-)** |
| peripheral benzodiazepine receptor | D21207 |  | **0.25** | **1.3** | | | 0.080 |  | **1.4** | 0.121 | |  | **1.8±0.3** |
| cytochrome c oxidase polypeptide Vb (COX5B) | X53157 |  | **0.21** | **1.3** | | | 0.021 |  | **1.3** | 0.031 | |  |  |
| glyceraldehyde-3-phosphate dehydrogenase (G3PDH; GADPH) | M32599 |  | **0.18** | **1.2** | | | 0.035 |  | **1.2** | 0.039 | |  |  |
| adipocyte protein aP2 | K02109 |  | **0.14** | **1.2** | | | 0.072 |  | **1.2** | 0.082 | |  |  |
| eukaryotic translation initiation factor 4E | M61731 |  | **-0.18** | **0.8** | | | 0.078 |  | **0.8** | 0.087 | |  |  |
| mitochondrial malate dehydrogenase | M16229 |  | **-0.19** | **0.8** | | | 0.048 |  | **0.8** | 0.061 | |  |  |
| cardiac/slow skeletal troponin C | M29793 |  | **-0.24** | **0.8** | | | 0.096 |  | **0.7** | 0.093 | |  |  |
| fast skeletal troponin C | M57590 |  | **-0.30** | **0.7** | | | 0.078 |  | **0.7** | 0.106 | |  |  |
| high-mobility group protein 4 | AF022465 |  | **-0.30** | **0.7** | | | 0.041 |  | **0.7** | 0.060 | |  |  |
| cytochrome c oxidase polypeptide VIIa3 (COX7A3) | AF037371 |  | **-0.33** | **0.7** | | | 0.015 |  | **0.7** | 0.021 | |  |  |
| anti-oxidant protein 1 | M28723 |  | **-0.33** | **0.7** | | | 0.011 |  | **0.7** | 0.016 | |  |  |
| natriuretic peptide precursor type B | S58667 |  | **-0.34** | **0.7** | | | 0.009 |  | **0.7** | 0.016 | |  |  |
| serine proteinase inhibitor 3 (SPI3) | U25844 |  | **-0.34** | **0.7** | | | 0.028 |  | **0.6** | 0.046 | |  |  |
| cardiac troponin T2 | L47549 |  | **-0.34** | **0.7** | | | 0.005 |  | **0.7** | 0.008 | |  |  |
| cardiac troponin I | U09181 |  | **-0.37** | **0.6** | | | 0.026 |  | **0.6** | 0.042 | |  |  |
| HSP60 | X55023 |  | **-0.38** | **0.6** | | | 0.040 |  | **0.6** | 0.052 | |  |  |
| cathepsin S precursor | AJ223208 |  | **-0.39** | **0.6** | | | 0.022 |  | **0.6** | 0.036 | |  |  |
| plasma selenoprotein P 1 | X99807 |  | **-0.39** | **0.6** | | | 0.021 |  | **0.6** | 0.048 | |  |  |
| mitochondrial glutamate oxaloacetate transaminase 2 | J02622 |  | **-0.41** | **0.6** | | | 0.012 |  | **0.6** | 0.020 | |  |  |
| medium-chain acetyl-Coenzyme A dehydrogenase | U07159 |  | **-0.42** | **0.6** | | | 0.007 |  | **0.6** | 0.012 | |  |  |
| mitochondrial aldehyde dehydrogenase 2 | U07235 |  | **-0.42** | **0.6** | | | 0.005 |  | **0.6** | 0.012 | |  |  |
| tubulin alpha 7 | M13443 |  | **-0.43** | **0.6** | | | 0.020 |  | **0.6** | 0.027 | |  |  |
| tropomodulin 1 | S76831 |  | **-0.48** | **0.5** | | | 0.013 |  | **0.5** | 0.031 | |  |  |
| anti-oxidant protein 2 | AF004670 |  | **-0.56** | **0.4** | | | 0.042 |  | **0.4** | 0.064 | |  |  |
| cytochrome b-245 alpha polypeptide | M31775 |  | **0.46** | **1.8** | | | 0.148 |  | **1.9** | 0.196 | |  | **2.1±0.4** |
| CD 81 antigen | X59047 |  | **0.19** | **1.2** | | | 0.139 |  | **1.3** | 0.149 | |  | **0.8±0.1** |
| basic fibroblast growth factor receptor 1 precursor (BFGF-R; FGFR1); FLG | X51893 |  | **0.16** | **1.2** | | | 0.729 |  | **1.3** | 0.722 | |  | **1.5±0.3** |
| prosaposin | U27340 |  | **0.15** | **1.2** | | | 0.374 |  | **1.2** | 0.362 | |  | **1.4±0.3** |
| cytochrome c oxidase polypeptide VIIc (COX7C) | X52940 |  | **0.03** | **1.0** | | | 0.853 |  | **1.0** | 0.787 | |  | **1.0±0.2** |
| CD 36 antigen | L23108 |  | **-0.15** | **0.9** | | | 0.496 |  | **0.8** | 0.494 | |  | **0.8±0.2** |
| interleukin 9 receptor | M84746 |  | **-0.16** | **0.8** | | | 0.622 |  | **0.7** | 0.542 | |  | **0.9(-)** |
| osteoglycin | D31951 |  | **-0.27** | **0.7** | | | 0.238 |  | **0.7** | 0.248 | |  | **0.7±0.1** |
