# Supplementary material for: Expression profiling of laser-microdissected intrapulmonary arteries in hypoxia-induced pulmonary hypertension
Source: Respir Res. 2005 Sep 19;6(1):109. doi: 10.1186/1465-9921-6-109 (PMC1261535; doi:10.1186/1465-9921-6-109)
Supplement: Additional File 3 — List of genes up- or down-regulated at day 21 of hypoxia. For changes in transcript abundance, the normalized difference D was used as a measure (see Methods). The D derived Q(D) is given and compared to the commonly used ratio of the intensities Q = IH/IN. If either intensity equals 0, log2(Q) cannot be determined meaningfully, whereas D gives -1 or +1 in these situations. This allows to include genes with zero values (i.e., "on" and "off" regulation) into further statistical analyses. In order to screen for relevant genes, the difference from zero of the D values was tested by a two-sided one-sample t-test. Those genes with p-values ≤ 0.1 were considered to be potentially regulated as real-time PCR confirmed in >90% the regulation. TaqMan PCR derived ratios are given as mean ± standard error of mean (SEM). [file 1465-9921-6-109-S3.doc]

| **Gen** | **Genbank** |  | | **Adjusted Difference: D** | | | | | |  | | **Adjusted Ratio: Q** | | | |  | | **TaqMan: Q** |
| --- | --- | --- | --- | --- | --- | --- | --- | --- | --- | --- | --- | --- | --- | --- | --- | --- | --- | --- |
|  | **ID** |  | | **Mean** | | **Q(D)** | | **p** | |  | | **Mean** | | **p** | |  | | **Mean±sem** |
| adipocyte complement related protein of 30 kDa | U37222 |  | **0.91** | | **10.8** | | 0.024 | |  | | **11.6** | | 0.100 | |  | |  | |
| adipsin | M11768 |  | **0.72** | | **3.6** | | 0.038 | |  | | **5.3** | | 0.135 | |  | |  | |
| procollagen 5 alpha 2 subunit (COL5A2) | L02918 |  | **0.70** | | **3.4** | | 0.074 | |  | | **3.5** | | 0.140 | |  | |  | |
| aldolase C isoform | S72537 |  | **0.70** | | **3.3** | | 0.013 | |  | | **3.3** | | 0.025 | |  | |  | |
| procollagen 1 alpha 1 subunit | U08020 |  | **0.66** | | **2.9** | | 0.019 | |  | | **3.7** | | 0.040 | |  | | **2.5**±**0.4** | |
| desmin | L22550 |  | **0.63** | | **2.7** | | 0.089 | |  | | **2.8** | | 0.150 | |  | |  | |
| matrix gamma-carboxyglutamate protein | D00613 |  | **0.62** | | **2.7** | | 0.007 | |  | | **2.9** | | 0.016 | |  | | **2.1**±**0.5** | |
| small inducible cytokine A21A precursor | U88322 |  | **0.59** | | **2.4** | | 0.046 | |  | | **2.8** | | 0.136 | |  | |  | |
| procollagen 1 alpha 2 subunit (COL1A2) | X58251 |  | **0.59** | | **2.4** | | 0.026 | |  | | **2.8** | | 0.038 | |  | | **2.3**±**0.2** | |
| carbonic anhydrase 3 | M27796 |  | **0.57** | | **2.3** | | 0.042 | |  | | **2.9** | | 0.068 | |  | | **3.4**±**0.7** | |
| annexin A3 | AJ001633 |  | **0.55** | | **2.2** | | 0.007 | |  | | **2.3** | | 0.018 | |  | |  | |
| stromal cell derived factor 3 | D50460 |  | **0.50** | | **2.0** | | 0.022 | |  | | **2.2** | | 0.071 | |  | |  | |
| non-muscle myosin light chain 3 (MLC3NM; MYLN): MYL6 | U04443 |  | **0.49** | | **2.0** | | 0.081 | |  | | **2.1** | | 0.120 | |  | | **1.3**±**0.4** | |
| endoplasmic reticulum protein | M73329 |  | **0.48** | | **1.9** | | 0.080 | |  | | **1.9** | | 0.111 | |  | |  | |
| cytoplasmic beta-actin (ACTB) | M12481 |  | **0.46** | | **1.8** | | 0.063 | |  | | **1.9** | | 0.087 | |  | |  | |
| cytochrome c oxidase polypeptide VI a1 (COX6A1) | L06465 |  | **0.44** | | **1.8** | | 0.008 | |  | | **1.8** | | 0.016 | |  | |  | |
| procollagen 3 alpha 1 subunit | X52046 |  | **0.39** | | **1.6** | | 0.058 | |  | | **1.8** | | 0.083 | |  | | **2.3**±**0.5** | |
| FK506 binding protein 1a (12 kDa) | X60203 |  | **0.38** | | **1.6** | | 0.096 | |  | | **1.6** | | 0.120 | |  | | **10.4**±**3.6** | |
| peripheral benzodiazepine receptor | D21207 |  | **0.35** | | **1.5** | | 0.044 | |  | | **1.6** | | 0.062 | |  | | **2.1**±**0.8** | |
| tubulin alpha 7 | M13443 |  | **0.29** | | **1.4** | | <0.001 | |  | | **1.4** | | <0.001 | |  | |  | |
| cytochrome c oxidase polypeptide VIIIa (COX8A) | U37721 |  | **0.28** | | **1.4** | | 0.048 | |  | | **1.4** | | 0.062 | |  | |  | |
| brain creatine kinase | M74149 |  | **0.28** | | **1.4** | | 0.079 | |  | | **1.4** | | 0.100 | |  | |  | |
| cytochrome b-245 alpha polypeptide | M31775 |  | **0.25** | | **1.3** | | 0.081 | |  | | **1.4** | | 0.121 | |  | | **1.4**±**0.3** | |
| adipocyte protein aP2 | K02109 |  | **0.21** | | **1.3** | | 0.095 | |  | | **1.3** | | 0.107 | |  | |  | |
| carboxypeptidase E | X61232 |  | **0.20** | | **1.3** | | 0.025 | |  | | **1.3** | | 0.030 | |  | |  | |
| olfactory receptor B7 | AF102520 |  | **-0.18** | | **0.8** | | 0.084 | |  | | **0.8** | | 0.098 | |  | |  | |
| voltage-dependent anion channel 2 | U30838 |  | **-0.22** | | **0.8** | | 0.082 | |  | | **0.8** | | 0.096 | |  | |  | |
| capping protein alpha 2 | U16741 |  | **-0.32** | | **0.7** | | 0.060 | |  | | **0.7** | | 0.072 | |  | |  | |
| heat shock protein cognate 70 | U27129 |  | **-0.36** | | **0.6** | | 0.100 | |  | | **0.6** | | 0.127 | |  | |  | |
| cathepsin D | X52886 |  | **-0.38** | | **0.6** | | 0.037 | |  | | **0.6** | | 0.051 | |  | |  | |
| Sjogren syndrome antigen B | L00993 |  | **-0.41** | | **0.6** | | 0.097 | |  | | **0.6** | | 0.145 | |  | |  | |
| interleukin 9 receptor | M84746 |  | **-0.42** | | **0.6** | | 0.002 | |  | | **0.6** | | 0.004 | |  | |  | |
| inhibitor of DNA binding 1 | M31885 |  | **-0.43** | | **0.6** | | 0.029 | |  | | **0.5** | | 0.040 | |  | |  | |
| dutt1 protein | Y17793 |  | **-0.46** | | **0.5** | | 0.082 | |  | | **0.5** | | 0.113 | |  | |  | |
| interleukin 10 receptor beta ( IL10R- beta; IL10RB) | U53696 |  | **-0.49** | | **0.5** | | 0.018 | |  | | **0.5** | | 0.035 | |  | |  | |
| high mobility group protein I | J04179 |  | **-0.53** | | **0.5** | | 0.019 | |  | | **0.5** | | 0.043 | |  | |  | |
| glucagon receptor | L38613 |  | **-0.54** | | **0.5** | | 0.004 | |  | | **0.5** | | 0.009 | |  | |  | |
| interleukin 10 receptor alpha precursor ( IL10R- alpha; IL10RA) | L12120 |  | **-0.57** | | **0.4** | | 0.018 | |  | | **0.4** | | 0.048 | |  | |  | |
| high mobility group protein 2 | Z46757 |  | **-0.60** | | **0.4** | | 0.050 | |  | | **0.3** | | 0.145 | |  | |  | |
| CD 36 antigen | L23108 |  | **-0.63** | | **0.4** | | 0.001 | |  | | **0.4** | | 0.006 | |  | | **0.4**±**0.1** | |
| Burkitt lymphoma receptor 1 | X71788 |  | **-0.68** | | **0.3** | | 0.085 | |  | | **0.3** | | 0.154 | |  | |  | |
| CC chemokine receptor type 5 (CMKBR5; CCCKR5; CCR5) | D83648 |  | **-0.90** | | **0.1** | | 0.016 | |  | | **0.1** | | 0.064 | |  | |  | |
| S100 calcium-binding protein A4 | D00208 |  | **0.77** | | **4.5** | | n.d | |  | | **4.5** | | n.d | |  | | **6.0±3.0** | |
| prosaposin | U27340 |  | **0.16** | | **1.2** | | 0.408 | |  | | **1.2** | | 0.385 | |  | | **3.5**±**0.7** | |
| CD 81 antigen | X59047 |  | **-0.13** | | **0.9** | | 0.535 | |  | | **0.8** | | 0.521 | |  | | **0.9**±**0.1** | |
